# Upregulation of Receptor Tyrosine Kinase Activity and Stemness as Resistance Mechanisms to Akt Inhibitors in Breast Cancer

**DOI:** 10.3390/cancers14205006

**Published:** 2022-10-13

**Authors:** Tiffany Tsang, Qingling He, Emily B. Cohen, Casey Stottrup, Evan C. Lien, Huiqi Zhang, C. Geoffrey Lau, Y. Rebecca Chin

**Affiliations:** 1 Department of Pathology, Beth Israel Deaconess Medical Center, Harvard Medical School, Boston, MA 02215, USA; 2 Tung Biomedical Sciences Centre, Department of Biomedical Sciences, City University of Hong Kong, Hong Kong; 3 Department of Neuroscience, City University of Hong Kong, Hong Kong; 4 Key Laboratory of Biochip Technology, Biotech and Health Centre, City University of Hong Kong Shenzhen Research Institute, Shenzhen 518057, China

**Keywords:** breast cancer, Akt, cancer stemness, drug resistance

## Abstract

**Simple Summary:**

Acquired resistance of cancer cells to targeted therapy poses a major clinical problem. Hyperactivation of the PI3K/Akt pathway is common in human malignancies, and numerous Akt inhibitors are undergoing clinical evaluation. However, mechanisms of acquired resistance to Akt inhibitors have not been studied extensively. By using phospho-RTK arrays, we demonstrated hyper-phosphorylation of multiple RTKs in Akt-inhibitor-resistant breast cancer cells. We further showed that the EGFR inhibitor could overcome Akt inhibitor resistance. In addition, our study revealed enhanced cancer stemness in resistant cells, and RNA sequencing identified several stem cell regulators that may contribute to acquired resistance.

**Abstract:**

The PI3K/Akt pathway is frequently deregulated in human cancers, and multiple Akt inhibitors are currently under clinical evaluation. Based on the experience from other molecular targeted therapies, however, it is likely that acquired resistance will be developed in patients treated with Akt inhibitors. We established breast cancer models of acquired resistance by prolonged treatment of cells with allosteric or ATP-competitive Akt inhibitors. Phospho-Receptor tyrosine kinase (Phospho-RTK) arrays revealed hyper-phosphorylation of multiple RTKS, including EGFR, Her2, HFGR, EhpB3 and ROR1, in Akt-inhibitor-resistant cells. Importantly, resistance can be overcome by treatment with an EGFR inhibitor. We further showed that cancer stem cells (CSCs) are enriched in breast tumor cells that have developed resistance to Akt inhibitors. Several candidates of CSC regulators, such as ID4, are identified by RNA sequencing. Cosmic analysis indicated that sensitivity of tumor cells to Akt inhibitors can be predicted by ID4 and stem cell/epithelial–mesenchymal transition pathway targets. These findings indicate the potential of targeting the EGFR pathway and CSC program to circumvent Akt inhibitor resistance in breast cancer.

## 1. Introduction

The phosphoinositide 3-kinase (PI3K)/Akt signaling pathway is among the most frequently dysregulated pathways in human cancers. Approximately 70% of breast cancers have hyperactive PI3K/Akt signaling [1,2]. The PI3K/Akt pathway can be activated in tumor cells through mutations or amplification of PIK3CA, loss of PTEN function and/or overexpression of Akt1, Akt2 and Akt3 [3]. Akt, being the central node of multiple signaling cascades, regulates cellular processes including proliferation, survival, metabolism and invasive migration [1]. Based on this premise, a number of Akt inhibitors have been developed and evaluated in the past decade as cancer therapeutic drugs for different solid tumors [4]. Broadly, the two major classes of Akt inhibitors are allosteric (e.g., MK2206) and ATP-competitive inhibitors (e.g., ipatasertib/GDC0068, capivasertib/AZD5363). As monotherapy, Akt inhibitors show modest responses in clinical trials. However, when using Akt inhibitors in combination strategies, promising results are emerging in recent trials [4]. For example, good clinical activity was observed with the combination of capivasertib and paclitaxel in patients with metastatic triple-negative breast cancer (TNBC) in the Phase II PAKT clinical trial [5]. This combination is currently being evaluated in Phase III trials. In another trial, combination of capivasertib and fulvestrant showed clinically meaningful activity in patients with Akt1 E17K-mutant, ER-positive metastatic breast cancer [6]. Akt inhibitors have also been combined with various targeted therapeutic agents. There is an ongoing Phase Ib trial (TAKTIC) for patients with metastatic breast cancer, evaluating the efficacy and toxicity of triplet combination of ipatasertib, CDK4/6 inhibitor palbociclib and fulvestrant. Indeed, just for breast cancer alone, more than 20 clinical trials are ongoing to test the efficacy of various Akt inhibitors. Given the active pursuit of Akt inhibitors in cancer drug development, it is important to identify the molecular mechanisms of anticipating acquired resistance, a major clinical problem for targeted therapies.

Some resistance mechanisms have been described for PI3K/Akt pathway inhibitors. In breast cancer, p110β activation has been shown to compensate for p110α-specific inhibition [7,8]. In addition, it is demonstrated that NEK9 and MAP2K4 play important roles in mediating resistance to the pan-PI3K inhibitor in TNBC [9]. Enhanced estrogen receptor, Notch, RSK3/4 and Myc pathways are also implicated in PI3K inhibitor resistance in different breast cancer contexts [10,11,12,13]. Acquired resistance mechanisms to Akt inhibitors, on the other hand, have not been studied extensively, in part being hampered by the lack of serial biopsy tumor materials. Nevertheless, it is shown that breast tumor cells acutely treated with Akt inhibitors resulted in expression and phosphorylation of different receptor tyrosine kinases (RTKs) including IGF1R, Her3 and insulin receptor [14,15]. To model acquired resistance in breast cancer, we previously generated MK2206-resistant lines, and showed a prominent role of Akt3 in mediating resistance [16]. In this study, we performed RTK arrays and RNA sequencing on different breast tumor lines with prolonged treatment of ATP-competitive or allosteric Akt inhibitors, with the goal of utilizing unbiased platforms to delineate pathways and proteins associated with acquired resistance. The catalog of changes in resistant cells was then correlated with observed phenotypes. RTK arrays revealed upregulation of multiple RTKs in Akt-inhibitor-resistant cells. We further showed that the EGFR inhibitor confers drug sensitivity to the Akt inhibitor. The resistant cells also displayed enhanced cancer stem cell (CSC) phenotypes, and several CSC-related candidates were identified with RNA sequencing. Dissecting the spectrum of resistance mechanisms may aid the development of therapeutic strategies to treat resistant breast tumors.

## 2. Materials and Methods

**Cell Culture.** T47D, BT474, MDA-MB-231, and MDA-MB-468 cells were obtained from American Type Culture Collection (ATCC, Manassas, VA, USA) and cultured according to their recommendation. MCF10-DCIS cells were obtained by Kornelia Polyak (Harvard Medical School, Boston, MA, USA). MCF10-DCIS was maintained in DMEM/F-12 (Gibco, Grand Island, NY, USA) supplemented with 5% horse serum (Gibco, Waltham, MA, USA), 20 ng/mL EGF (R&D, Minneapolis, MN, USA), 10 µg/mL Insulin (ThermoFisher Scientific, Waltham, MA, USA), 100 ng/mL final cholera toxin (List Biological laboratories, Campbell, CA, USA) and 500 ng/mL hydrocortisone (Sigma, Burlington, MA, USA). All cell lines were passaged for < 6 months, and routinely assayed for mycoplasma contamination. 

**AKT-inhibitor-resistant line generation.** Akt-inhibitor-resistant BT474 cell lines GDC0.2-1.8 and MK0.2-1.8 were generated by gradual dose escalation of Akt inhibitor (GDC0068 or MK2206) from 0.2 μM to 1.8 μM for a period of 3 months, then the cells were maintained in 1.8 μM of respective inhibitor. BT474 GDC1-2 resistant line was generated by culturing cells in increasing concentration of GDC0068 from 1 μM to 2 μM for 3 months. T47D MK5 and GDC5 resistant lines were generated by exposing cells chronically to 5 μM MK2206 or GDC0068 for 3 months. Parental cells were cultured in the presence of dimethyl sulfoxide (DMSO) (Sigma, St. Louis, MO, USA). Akt inhibitor was replaced every 3–4 days. Cells were considered resistant when they could be cultured routinely in the presence of high dose of Akt inhibitors. MK2206 and GDC0068 (Selleck Chemical, Houston, TX, USA) have been described previously [17,18].

**Antibodies.** All primary antibodies in this study except p85 and ID4 were obtained from Cell Signaling Technology (CST, Beverly, MA, USA). Anti-p85 polyclonal antibody was generated in-house and has been described [19]. ID4 antibody (clone DML07) was purchased from Millipore (MilliporeSigma, Carlsbad, CA, USA). PE-conjugated anti-CD44 antibody was obtained from BD Biosciences (Franklin Lakes, NJ, USA). Horseradish peroxidase-conjugated anti-mouse and anti-rabbit immunoglobulin G (IgG) antibody were purchased from Chemicon (Chemicon International, Temecula, CA, USA). Anti-rabbit Dylight650 antibody was obtained from Jackson laboratory (Bar Harbor, ME, USA).

**Cell viability assays.** Cells were seeded into 96-well plates (Corning, Oneonta, NY, USA) at a density of 5000 cells per well. Then, 24 hours later, cells were treated with inhibitor for 48 h. Cell viability was measured by the WST-1 assay (Clontech, San Jose, CA, USA) according to manufacturer’s protocol.

**Mammosphere formation assay.** Cells (1000–2500 cells per well) were seeded to ultra-low attachment 6-well plates (Corning, Oneonta, NY, USA, 3471) and cultured in mammosphere medium containing DMEM/F12 supplemented with B27 (Gibco, Grand Island, NY, USA, 12587010) and 20 ng/mL EGF (R&D, Minneapolis, MN, USA, 236-EG) for 5–6 days to form 1st generation mammosphere. Nikon Eclipse Tis2 microscope (Nikon corporation, Minato City, Tokyo, Japan) was used to capture images of mammospheres. Number of mammospheres with diameter ≥ 70 µm were counted using the Nikon NIS-Elements D software (Nikon corporation, Minato City, Tokyo, Japan). Mammosphere formation efficiency (MFE) is calculated with formula: # mammospheres/# cells seeded * 100%. Second- (2°) and third-generation (3°) mammosphere formation assays were performed as previously described [20]. Briefly, mammospheres from 1st generation were collected and dissociated by trypsin. All cells were then seeded to a new ultra-low attachment plate with similar densities and cultured for 5–6 days. 

**Flow cytometry analysis.** Trypsinized cells were collected by centrifugation at 800× *g* for 5 min, and then stained with PE-CD44 antibody on ice for 30 min. After washing with PBS 3 times, cells were fixed with 2% formaldehyde/PBS for 15 min. Permeabilization was carried out for 2 min using 0.5% TritonX/PBS. Cells were stained with anti-Slug antibody in binding buffer for 30 min, followed by washing with PBS 3 times. Cells were then incubated with Anti-rabbit Dylight650 antibody for 30 min and washed with PBS 3 times. Flow cytometry analysis was performed with FACSCalibur (Becton-Dickinson, Franklin Lakes, NJ, USA) and FlowJo (BD Biosciences, Ashland, OR, USA) software.

**Human Phospho-RTK array.** Phospho-RTK arrays were performed using Proteome Profiler Human Phospho-RTK array kit (R&D Systems, Minneapolis, MN, USA, ARY001B) according to the manufacturer’s protocol. This array allows the screening of 49 different phosphorylated RTKs simultaneously. Briefly, after blocking the array with Array Buffer I for 1 hour, 300 µg protein lysates were incubated with the array at 4 °C for 16 h, which allowed binding of both phosphorylated and unphosphorylated RTKs. Arrays were washed 3 times with Wash Buffer, followed by incubation with anti-phospho-tyrosine-HRP detection antibody for 2 h. After washing the arrays 3 times, they were developed using enhanced chemiluminescence substrate (Pierce, Rockford, IL, USA). 

**RNA sequencing.** Total RNA of parental and Akt-inhibitor-resistant cells was extracted using RNeasy kit (Qiagen, Germantown, MD, USA) according to manufacturer’s manual. The RNA library construction and RNA-seq analysis was performed by BGI Americas Company (Cambridge, MA, USA). Experiment was performed without biological replicates. The libraries were multiplexed and sequenced on Hiseq 2000 (Illumina, San Diego, CA, USA) with read length of paired-end 50 base pairs. Gene expression raw counts were computed, where genes with at least 1 count per million reads (CPM) in at least 1 library were included. For differential expression analysis, multi-dimensional scaling was performed and showed clear clustering of different cell lines. Pair-wise comparison of all genes across samples was then performed. LogFC was used for cutoff for differentially expressed genes. Genes that have at least two-fold change (logFC > 1 or logFC < −1) were defined as differentially expressed. DAVID Bioinformatics Resources was used to perform gene set enrichment analysis (GSEA). Benjamini value of 1 × 10^−3^ was used as the cutoff to determine if the term/pathway was significantly enriched.

**siRNA transfection.** MDA-MB-468 cells were plated to 6-well plates (3 × 10^5^/well) and allowed to grow for 24 h before transfection. Cells were transfected with ID4 or control siRNAs (ON-TARGETplus Human ID4 siRNA-SMARTpool, Dharmacon, Lafayette, CO, USA) using Lipofectamine RNAiMAX Transfection Reagent (Invitrogen, Waltham, MA, USA) according to the manual. In brief, Lipofectamine RNAiMAX Transfection Reagent (final 7.5 μ/well) and siRNAs (final 25 pmol/well) were diluted in 150 μL of Opti-MEM (Gibco, Grand Island, NY, USA) separately. Diluted siRNAs and diluted Lipofectamine RNAiMAX Transfection Reagent (1:1 ratio) were mixed and incubated for 5 min at room temperature. siRNA–lipid complexes were added to cells, which were cultured for 3 days for gene knockdown.

**Quantitative real-time RT-PCR.** Total RNA was isolated with RNeasy Mini Kit (Qiagen, Germantown, MD, USA). Reverse transcription was performed using multiscribe reverse transcriptase and random hexamers (Applied Biosystems, Foster City, CA, USA). Quantitative real-time PCR was performed using an ABI Prism7700 sequence detector (Applied Biosystems, Foster City, CA, USA). ID4 primer: sense, 5′-TTGGCCTGGCTCTTAATTTG-3′; anti-sense, 5′-TGCAATCATGCAAGACCACT-3′; Slug primer: sense, 5′-CTGGGCGCCCTGAACATGCAT-3′; anti-sense, 5′-GGCTTCTCCCCCGTGTGAGTTCTA-3′; FOXK1 primer: sense, 5′-ACACGTCTGGAGGAGACAGC-3′; anti-sense, 5′-GAGAGGTTGTGCCGGATAGA-3′; SNAIL primer: sense, 5′-CGAGTGGTTCTTCTGCGCTA-3′; anti-sense, 5′-CTGCTGGAAGGTAAACTCTGGA-3′; NOTCH3 primer: sense, 5′-GTGGCCCTCATGGTATCTGC-3′; anti-sense, 5′-CATGGGTTGGGGTCACAGT-3′; TWIST1 primer: sense, 5′-CACGAGCGGCTCAGCTACGC-3′; anti-sense, 5′-ACAATGACATCTAGGTCTCCGGCCC-3′; SOX4 primer: sense, 5′-CCCAGCAAGAAGGCGAGTTA-3′; anti-sense, 5′-CATCGGCCAAATTCGTCACC-3′; IRF1 primer: sense, 5′-GAGGAGGTGAAAGACCAGAGCA-3′; anti-sense, 5′-TAGCATCTCGGCTGGACTTCGA-3′; BMP2 primer: sense, 5′-TGCACCAAGATGAACACAGC-3′; anti-sense, 5′-GTGCCACGATCCAGTCATTC-3′; TWIST2 primer: sense, 5′-TGCTCACTCCCGCCAACGTT-3′; anti-sense, 5′-GGCGCGCCAGGAGGAGATTCT-3′; GAPDH primer: sense, 5′-GCAAATTCCATGGCACCGT-3′; anti-sense, 5′-TCGCCCCACTTGATTTTGG-3′. PCR reactions were carried out in triplicate. Quantification of mRNA expression was calculated by the dCT method with GAPDH as the reference gene.

**Immunoblotting.** Cells were lysed in RIPA buffer (1% NP-40, 0.5% sodium deoxycholate (SDC), 0.1% SDS, proteinase inhibitor cocktail, 50 nM calyculin, 1 mM sodium pyrophosphate, 20 mM sodium fluoride, 150 mM NaCl, 50 mM Tris-HCl (pH 7.5)) (Chemicals purchased from Sigma (Burlington, MA, USA)) on ice for 15 min. Cell extracts were pre-cleared by centrifugation at 13,000 rpm at 4 °C for 10 min and protein concentration was measured by a Beckman Coulter DU-800 machine (Beckman Coulter, Brea, CA, USA) with the Bio-Rad protein assay reagent (Bio-Rad, Hercules, CA, USA). Lysates were then resolved by SDS-PAGE and the blots were incubated with the indicated antibodies. Signals were detected using enhanced chemiluminescence substrate (Pierce, Rockford, IL, USA). 

**Statistical Analysis.** Student’s *t* tests or ANOVA were used to determine statistical significance between conditions. In all figures, data are presented as mean ± standard error of the mean (SEM) for one representative experiment. Significance between conditions is denoted as *, *p* < 0.05; **, *p* < 0.01; ***, *p* < 0.001. For mammosphere analyses, all mammospheres in the well were counted, for 3 wells per condition.

## 3. Results

### 3.1. Generation and Characterization of Cells Resistant to Akt Inhibitors

**Figure 1:**
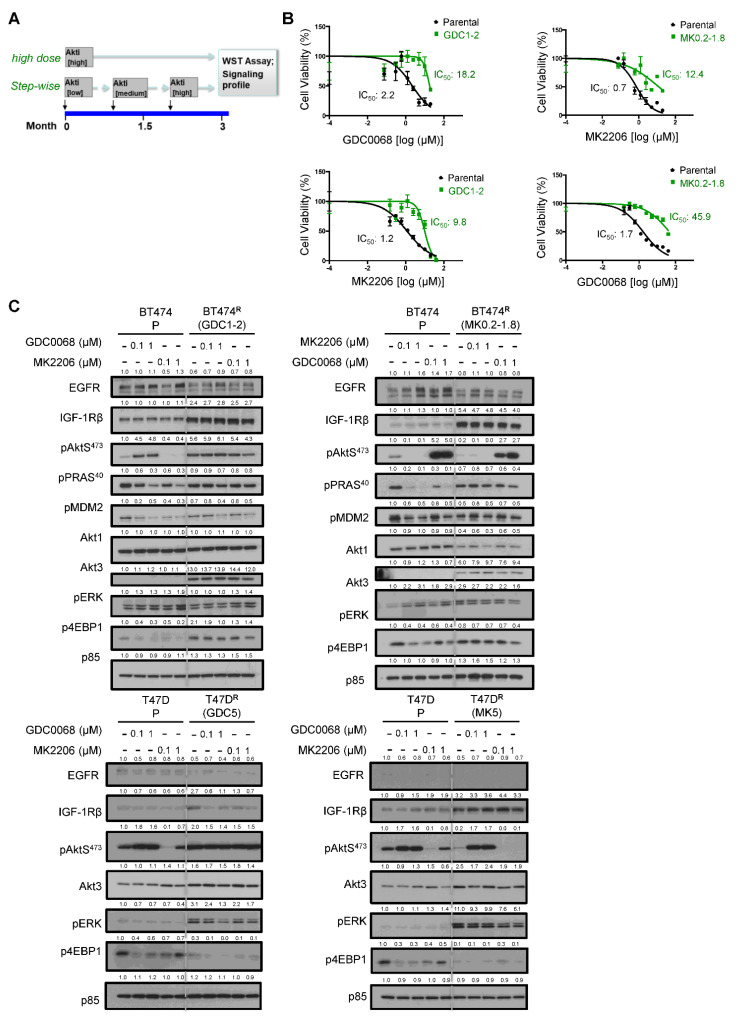
Establishment and characterization of breast tumor cell lines resistant to Akt inhibitors. (**A**) Schematics of establishing Akt-inhibitor-resistant lines using high dose or step-wise method. (**B**) BT474 parental (black) and Akt-inhibitor-resistant (green) cells were seeded to 96-well plates and then treated with MK2206 or GDC0068 for 48 h. Cell viability was assessed by WST assays and calculated relative to the untreated cells. Data, mean ± standard error of the mean (SEM) with n = 3. (**C**) Parental and Akt-inhibitor-resistant cells were seeded to plates without Akt inhibitor for 48 h, followed by treatment with Akt inhibitor for 1 h. Whole-cell lysates were subjected to immunoblotting; p85 was used as a loading control.

We first generated in vitro resistant breast tumor cells to Akt inhibitors, using a method previously described [16]. Two luminal breast tumor lines with a hyperactive PI3K/Akt pathway were chosen for modeling acquired resistance. BT474 and T47D are luminal B cells with Her2 overexpression and luminal A cells with activating *PIK3CA* mutation (H1047R), respectively. MK2206 was initially evaluated in clinical trials for breast cancer, and GDC0068 is currently being tested in combination with other therapeutic agents in various trials. Akt-inhibitor-resistant derivative BT474 lines were developed by chronically treating tumor cells with gradually increasing doses of inhibitor (starting at 0.2 μM or 1 μM) (Figure 1A). Three months later, cells became resistant to the Akt inhibitor in cell viability assays, with >8-fold increase in IC_50_ compared to parental lines (Figure 1B). The resistant pools of cells are termed BT474 R (GDC1-2; MK0.2-1.8; step-wise fashion). The BT474 R lines also show cross-resistance to the other class of Akt inhibitor. We then performed dose–response experiments and showed that the BT474 R lines are resistant to Akt inhibitors at multiple nodes of the PI3K/Akt pathway, including pAkt S^473^, pPRAS40 T^246^, pMDM2 S^166^ and p4EBP1 S^65^ (Figure 1C). Agreeing with our previous findings [16], Akt3 and IGF1R are dramatically upregulated in BT474 R cells. Indeed, the protein expression of Akt3 in resistant lines is de novo expression. Interestingly, whereas T47D R cells (GDC5; MK5; chronic high dose fashion) exhibit downregulation of p4EBP1 S^65^, these cells have significant upregulation of pERK, indicating the potential of T47D cells in utilizing the ERK pathway as a compensatory mechanism.

### 3.2. Activation of EGFR as a Mechanism of Acquired Resistance to AKT Inhibitors

**Figure 2:**
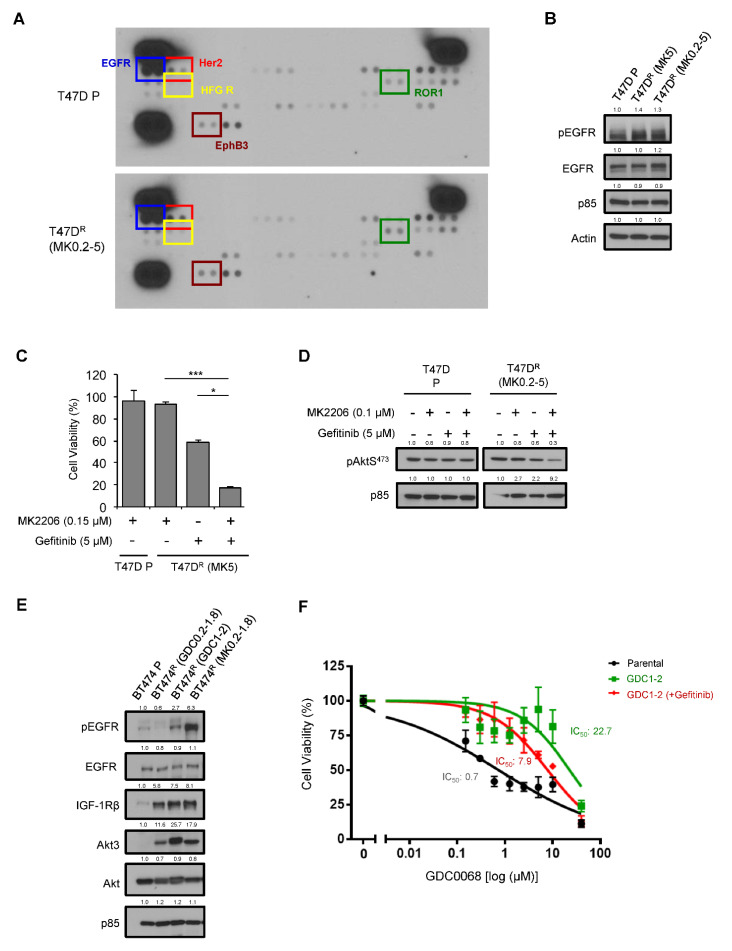
Upregulation of RTKs in Akt-inhibitor-resistant breast tumor cells. (**A**) Phospho-RTK arrays performed on lysates from T47D parental and resistant cells. (**B**) Western blot analysis on lysates of T47D parental and resistant cells. (**C**) T47D parental or resistant cells were seeded to plates without MK2206 for 24 h, followed by treatment with Gefitinib (5 μM) and/or MK2206 (0.15 μM) for 48 h. Cell viability was determined by WST assays. Data, mean ± SEM: *, *p* < 0.05; ***, *p* < 0.001 (ANOVA, n = 3). (**D**) T47D cells were seeded to plates without MK2206 for 48 h. Cells were then treated with Gefitinib (5 μM) for 1h, followed by MK2206 (0.15 μM) for 1 h. Whole-cell lysates were subjected to immunoblotting. (**E**) Western blot analysis on lysates of BT474 parental and resistant cells. (**F**) BT474 cells were seeded to plates without GDC0068 for 24 h. Cells were then treated with GDC0068 alone, or in combination with Gefitinib (5 μM) for 48 h, followed by WST assays. Data, mean ± SEM, n = 3.

To profile the activation of RTKs in resistant cells, we performed an RTK array which allowed us to simultaneously assess tyrosine phosphorylation of 49 human RTKs. Phosphorylation of EGFR, Her2, HFGR, EhpB3 and ROR1 were shown to be upregulated significantly in T47D R (MK0.2-5) cells (Figure 2A and Figure S1). There was no change in EGFR protein expression in T47D R cells (Figure 1C and Figure 2B), yet pEGFR levels increased, as evidenced in both the RTK array (Figure 2A) and immunoblots (Figure 2B). To determine if EGFR activation plays an important role in Akt inhibitor resistance, we treated T47D R cells with the EGFR inhibitor Gefitinib and/or MK2206. Whereas treating resistant cells with MK2206 and Gefitinib resulted in 7% and 41% reduction in cell viability, respectively, combination treatment led to 82% cell viability reduction (Figure 2C). Combination treatment also robustly reduced the phosphorylation of Akt in resistant cells but not parental cells (Figure 2D), suggesting that inhibiting EGFR could overcome Akt inhibitor resistance in T47D cells. We also examined the activation of EGFR in BT474 R lines. Similar to T47D R cells, we observed upregulation of pEGFR in BT474 R (GDC1-2 and MK0.2-1.8) lines without changes in EGFR protein expression (Figure 2E). Gefitinib treatment of BT474 R (GDC1-2) cells resulted in a 3-fold decrease in IC_50_ (Figure 2F). Taken together, these data suggest that EGFR mediates Akt inhibitor resistance in luminal breast cancer cells. 

### 3.3. Enhanced Cancer Stem Cell Properties in Akt-Inhibitor-Resistant Cells

**Figure 3:**
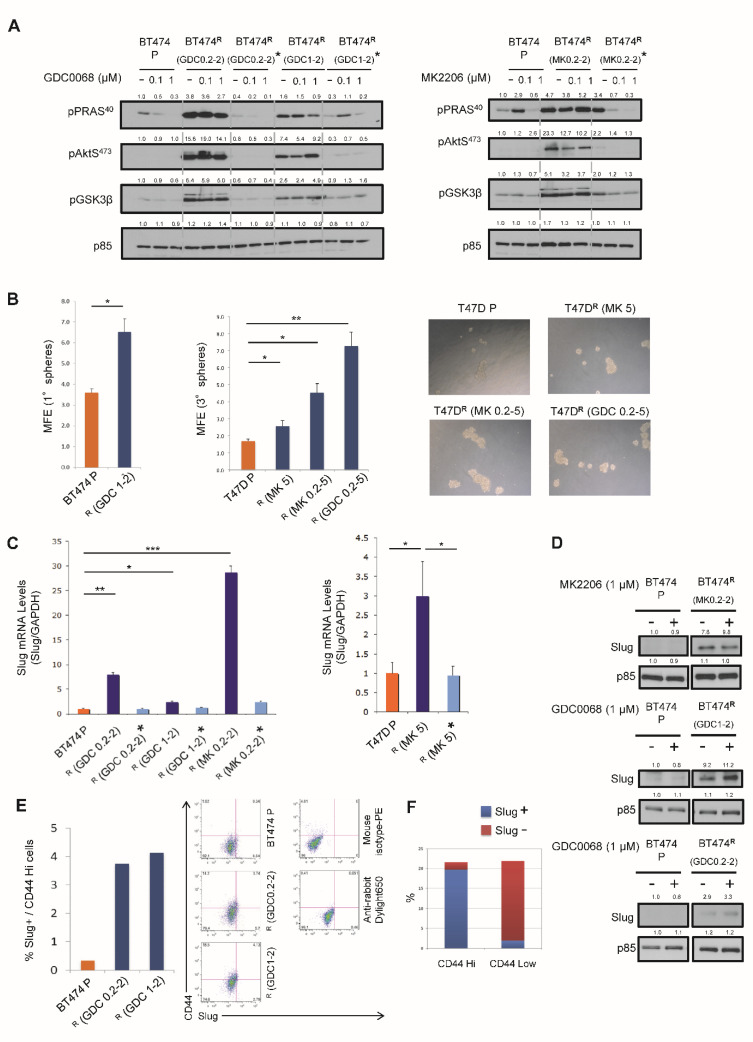
Upregulation of CSC phenotypes in Akt-inhibitor-resistant tumor cells. (**A**) BT474 parental and Akt-inhibitor-resistant cells were seeded to plates in the absence of Akt inhibitor for 48 h. Cells were then treated with MK2206 or GDC0068 for 1 h. Whole-cell lysates were subjected to immunoblotting. * Resistant cells were cultured in the absence of Akt inhibitor for 3.5 weeks, before Western blot analysis. (**B**) Mammosphere forming assays of parental and resistant cells. BT474 cells were seeded for first-generation (1°) mammosphere formation assay. T47D cells were seeded for first generation of mammosphere formation assay, followed by second and then third generation (3°). Mammosphere forming efficiency (MFE). Data, mean ± SEM: *, *p* < 0.05; **, *p* < 0.01 (ANOVA, n = 3). Morphology of mammospheres is shown in the representative phase-contrast images. (**C**) Quantification of Slug mRNA levels in parental and resistant cells by real time RT-PCR. * Akt inhibitor was removed from resistant lines for 3.5 weeks prior to analysis. Data, mean ± SEM: *, *p* < 0.05; **, *p* < 0.01; ***, *p* < 0.001 (ANOVA, n = 3). (**D**) Immunoblotting showing upregulation of Slug in various BT474 resistant lines. (**E**) FACS analysis to quantify % of Slug+/CD44 hi cells in BT474 parental and resistant cells. Original FACS data are shown on the right. (**F**) FACS analysis to assess co-expression of Slug and CD44 in breast MCF-10-DCIS tumor cells at the single-cell level.

We have previously reported that resistance phenotypes to Akt inhibitors in T47D cells are reversible [16]. Here, we also demonstrated the reversibility of signaling in BT474 R cells. The Akt inhibitor was removed from resistant lines for 24 days prior to signaling analysis. Whereas resistant cells did not display dose-dependent phosphorylation inhibition of PRAS40 and GSK3β by Akt inhibitors, upon drug removal, phosphorylation inhibition of Akt substrates was similar to those seen in parental cells (Figure 3A). We also examined reversibility of resistance to Akt inhibitors in a TNBC line MDA-MB-231. Compared to cells chronically treated with the Akt inhibitor GSK690693 (ATP-competitive inhibitor) for 3.5 months, resistant cells which had the drug removed for 1 month re-acquired sensitivity to the Akt inhibitor (Figure S2). However, when these cells were re-challenged with an Akt inhibitor for 1 week, they quickly regained resistance. These findings suggest that reversibility of Akt inhibitor resistance is a general phenomenon, regardless of the class of Akt inhibitors and breast cancer subtypes. Given the reversibility upon drug discontinuation, our previous observation of epithelial to mesenchymal transition (EMT) in Akt inhibitor resistance [16] and the link of EMT and cancer stemness [21], we next investigated if Akt inhibitor-resistant cells show enhanced cancer stem cell (CSC) properties. In mammosphere formation assays, increased mammosphere forming efficiency (MFE) was observed in both BT474 R and T47D R lines, compared to parental cells (Figure 3B). Interestingly, the mammospheres of BT474 R (MK0.2-2) were much smaller than the ones from the parental line, with diameters <70 µm, which preclude assessment of MFE in this resistant line. Slug is a transcription factor that regulates the mammary stem cell state [22]. In breast cancer, enhanced Slug expression is associated with poor patient prognosis [23]. Indeed, we recently identified Slug as a downstream target of YB1 in Akt3-mediated cancer stemness in TNBC [24]. Agreeing with the role of Slug in CSC properties, significant upregulation of Slug was found in resistant lines (Figure 3C). Upon drug removal for 3.5 weeks, Slug expression in the resistant cells decreased to a level comparable to the parental cells, suggesting that expression of Slug is regulated epigenetically. Increased protein levels of Slug were also observed in resistant cells (Figure 3D). Furthermore, using FACS analysis, we showed that Slug+/CD44^high^ CSCs are enriched in the resistant population compared to the parental line (Figure 3E). Indeed, Slug expression was found to correlate with CD44 levels in breast cancer cells at the single-cell level (Figure 3F).

**Figure 4:**
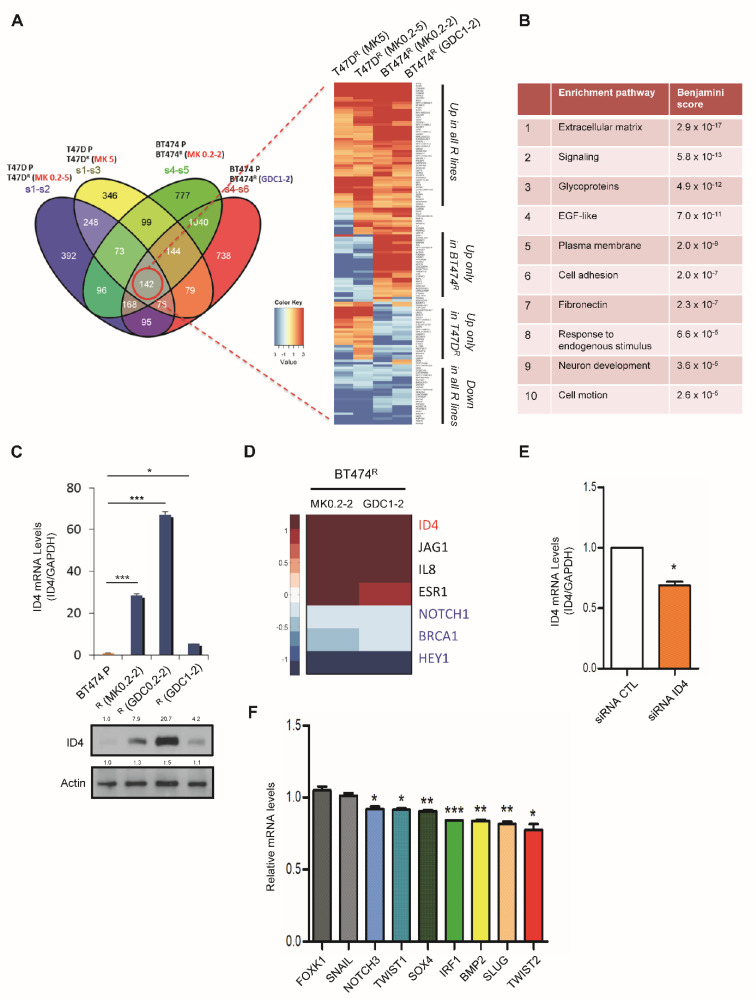
RNA sequencing reveals upregulation of ID4 in Akt-inhibitor-resistant cells. (**A**) Left: Venn diagram of distribution of differentially expressed genes in resistant lines. S1, T47D parental. S2, T47D MK0.2-5. S3, T47D MK5. S4, BT474 parental. S5, BT474 MK0.2-2. S6, BT474 GDC1-2. Right: Heatmap displaying genes (142) in which expression is changed (logFC > 1 or logFC < −1) across all comparisons; logFC of these genes are plotted in the heatmap. (**B**) Gene set enrichment analysis showing the top 10 enrichment pathways in T47D resistant cells. (**C**) Upregulation of ID4 in BT474 resistant cells at the mRNA (top) and protein (bottom) levels. Error bars, mean ± SEM: *, *p* < 0.05; ***, *p* < 0.001 (ANOVA, n = 3). (**D**). Heatmap showing alteration of ID4 pathway gene expression in the indicated BT474 resistant lines as compared to parental cells. (**E**). MDA-MB-468 cells were transfected with SMARTpool siRNAs of ID4 for 3 days, followed by qRT-PCR. Error bars, mean ± SEM: *, *p* < 0.05 (Student’s *t* test, n = 3). (**F**) Bar graphs depicting mRNA expression of stemness-related genes in ID4-knockdown cells compared to control cells. Error bars, mean ± SEM: *, *p* < 0.05; **, *p* < 0.01; ***, *p* < 0.001 (ANOVA, n = 3).

**Figure 5:**
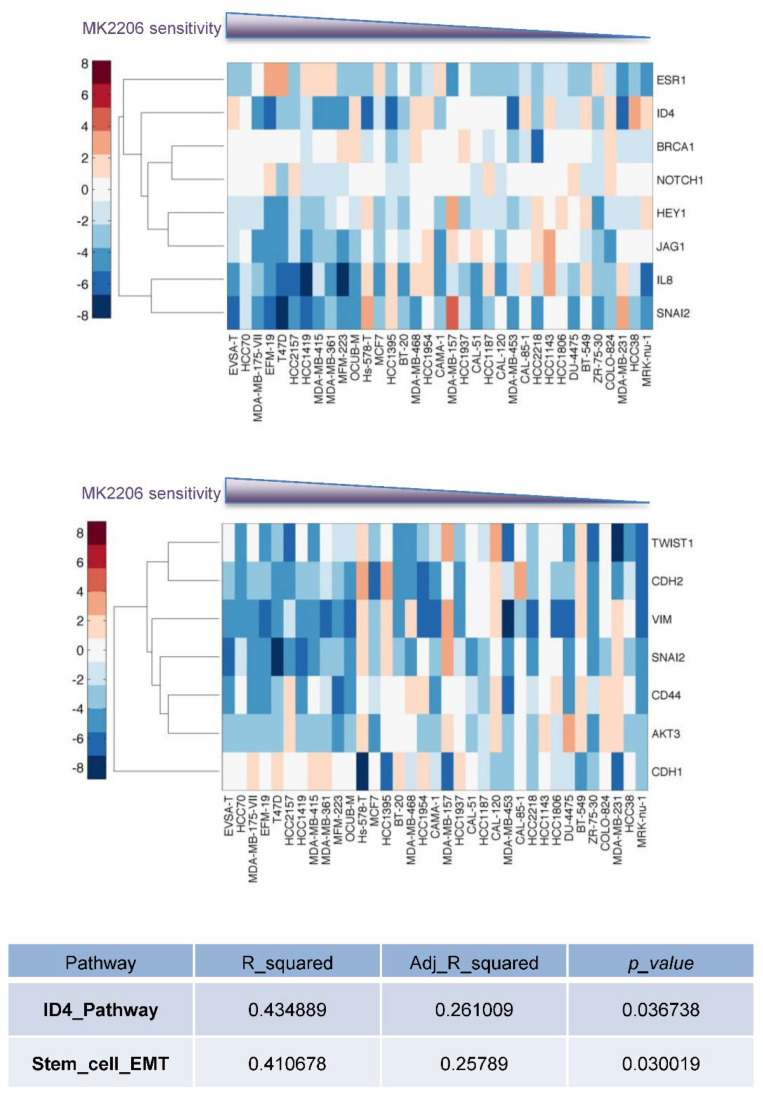
Genes in ID4 and stem cell/EMT pathways predict sensitivity to MK2206. Upper panel: Heatmap of pathway analysis showing relative expression of various genes in ID4 pathway in different breast tumor lines. Tumor lines are arranged based on MK2206 sensitivity. The cell line on the left (EVSA-T) is the most sensitive to MK2206. Lower panel: Heatmap showing relative expression of various genes in stem cell/EMT pathway. Table: Using cosmic analysis with multiple regression method, the data indicate that the expression values of each gene within the set (ID4, stem cell/EMT) are able to predict the MK2206 sensitivity for each cell line tested.

To identify genes and pathways that are involved in Akt resistance and promoting CSC phenotypes, we next performed RNA-seq to analyze the transcriptome of parental and Akt-inhibitor-resistant cells (T47D, BT474). A total of 142 protein-coding genes were either up- or downregulated (logFC > 1) in resistant lines (Figure 4A, Table S1). As expected, Akt3 and a few other genes in the PI3K/Akt pathway (e.g., HER3, insulin receptor substrate 2) were found to be upregulated (logFC > 0.5) in all resistant lines (Table S1). Gene set enrichment analysis revealed the five most highly enriched pathways in resistant cells including “extracellular matrix”, “signaling”, “glycoproteins”, “EGF-like” and “plasma membrane” (Figure 4B). Given the enhanced CSC properties we observed in resistant cells, we next validated a transcription factor, Inhibitor of DNA Binding 4 (ID4), one of the most highly upregulated genes in BT474 resistant lines. ID4 is a key regulator of mammary stem cells, and is associated with a CSC-like phenotype and poor prognosis of TNBC [25]. We demonstrated that ID4 is significantly upregulated in resistant lines at both mRNA and protein levels (Figure 4C). Known downstream targets of ID4 include Hey1, Notch1 and Brca1 [25,26]. Brca1 is downregulated in the resistant lines (Figure 4D), consistent with ID4 as a negative regulator of Brca1 [26]. Hey1 and Notch1 are members of the Notch pathway, which are upregulated under differentiation conditions [25]. In agreement with the enrichment of CSCs in our resistant lines, Hey1 and Notch1 were downregulated in the resistant cell lines (Figure 4D). To further examine the role of ID4 in cancer stemness, we knocked down ID4 in MDA-MB-468 breast tumor cells using SMARTpool siRNAs (Figure 4E), and tested the expression of a panel of stemness genes including Twist1, Twist2 [27], Snail, Slug [24], BMP2 [28], IRF1 [29], SOX4 [30], Foxk1 [31] and Notch3 [27]. Depletion of ID4 resulted in downregulation of most stemness-related genes we tested, including Twist2, Slug, BMP2, IRF1, SOX4, Twist1 and Notch3 (Figure 4F). Importantly, by performing cosmic analysis in 35 tumor lines, we found that the ID4 as well as stem cell/EMT pathway targets significantly predict sensitivity of tumor cells to MK2206 (ID4 pathway, *p* = 0.037; stem cell/EMT pathway, *p* = 0.030; Figure 5). These findings indicate that acquisition of Akt inhibitor resistance in breast tumor cells is accompanied by the CSC program, and our RNA-seq revealed genes that may contribute to Akt inhibitor resistance.

## 4. Discussion

Dysregulation of the PI3K/Akt pathway occurs at a high frequency in breast cancer, and a number of Akt inhibitors have been tested extensively in different clinical trials. Indeed, promising evidence of efficacy was observed in recent Phase II trials of Akt inhibition in TNBC [4]. In the LOTUS trial, combination treatment of ipatasertib and paclitaxel resulted in a 56% improvement in progression-free survival for patients with TNBC harboring PIK3CA/AKT1/PTEN alterations [32]. In addition to breast cancer, improved progression-free survival was also observed in a subgroup of prostate cancer patients receiving ipatasertib plus abiraterone in a Phase III clinical trial [33]. It is, however, anticipated that the long-term clinical efficacy will be challenged by the emergence of acquired resistance. Here, we sought to identify signaling and gene expression alterations that emerge upon chronic exposure to Akt inhibitors in two luminal breast tumor lines, using unbiased approaches including RTK arrays and RNA-seq. The ultimate goal is that by understanding the adaptive responses, vulnerability could be identified and provides rationale for innovative combination approaches [34]. For example, PI3K inhibitors have been shown to induce DNA damage in breast cancer cells [35]. Treating these cells with PARP inhibitors may offer an opportunity to induce synthetic lethality. In our study, using RTK arrays, we also identified several rewirings of signaling pathways in Akt-inhibitor-resistant cells. For instance, higher phosphorylation of EGFR, Her2, HGFR, EphB3 and ROR1 was observed in resistant cells. Interestingly, none of these RTKs showed consistent mRNA upregulation in our RNA sequencing studies. These observations suggest that RTK hyperactivation is via post-translational modification of RTKs, formation of novel RTK complexes and/or increased binding of their respective receptor ligands [34]. Importantly, we showed that treatment of Akt-inhibitor-resistant cells with Gefitinib sensitized them to the Akt inhibitor, suggesting potential strategy to overcome resistance to Akt inhibition in luminal breast cancer. In addition to EGFR, other RTKs upregulated in our array are implicated in targeted therapy resistance. For example, high levels of a truncated form of Her2 (p95-Her2) are shown to be responsible for mediating resistance to anti-Her2 therapy [36]. Point mutation and increased copy number of HGFR are also involved in the development of drug resistance in multiple cancers [37]. EphB3 induction was observed in EGFR-inhibitor-resistant colorectal cancer and FGFR-inhibitor-resistant gastric cancer [38,39]. Whether these RTK activities play important roles in conferring Akt inhibition in breast cancer awaits further investigation. It is noteworthy that upregulation of different RTKs can converge on common downstream effectors. Identifying such convergence signaling will offer therapeutic opportunities to combat resistance.

The reversibility of drug resistance in our model suggests that epigenetic mechanisms play an important role. We further showed that Akt-inhibitor-resistant cells exhibit enhanced CSC properties. This is in line with the thought that CSC phenotypes link to EMT [21], in which Akt-inhibitor-resistant lines display EMT properties, including downregulation of E-Cadherin, upregulation of N-Cadherin as well as Vimentin and increased invasiveness [16]. Indeed, CSCs have been implicated in drug resistance of a variety of cancers, including breast cancer [40,41,42], glioblastoma [43], colorectal cancer [44] and acute myelogenous leukemia [45]. To identify differentially expressed genes involved in CSC phenotypes and Akt inhibitor resistance, we performed RNA-seq in the resistant and parental lines. A number of genes in the PI3K/Akt pathway, including Akt3, IGF1R, Her3 and IRS2, were upregulated in the resistant cells. In addition to Akt3 and IGF1R [16], Her3 has been implicated in Akt-inhibitor-acquired resistance [14,15,46]. In search for CSC-related genes that are consistently upregulated in all resistant lines, we identified ID4 as a priority gene in our RNA-seq analysis and validated its significant upregulation in Akt-inhibitor-resistant cells, at both mRNA and protein levels. In TNBC, ID4 is specifically expressed in a subset of the population with a stem-like transcriptional profile [25]. ID4 has been shown to promote chemo-resistance and stemness of glioma cells by promoting SOX2 expression [47], but has yet to be studied in the context of breast cancer resistance. Mechanistically, ID4 promotes mammary stem cell properties by regulating Notch signaling and other pathways to repress commitment to the luminal fate. Upon overexpression of ID4, expression of luminal progenitor marker BRCA1 is reduced. In addition, ID4 negatively regulates Notch pathway genes, including Hey1 and Notch1 [25]. Our RNA-seq data indicated an upregulation of ID4 and downregulation of Notch 1, Hey1 and BRCA1 in Akt-inhibitor-resistant cells, which are in line with the known function of ID4 in suppressing the Notch pathway. Interestingly, forced expression of Slug in differentiated luminal cells dramatically increases the expression of ID4 and several other basal markers [23], suggesting that Slug not only is critical for inducing a basal-like state, but also regulates ID4 expression. Whether Slug regulates ID4 as well as its downstream targets and confers drug resistance in breast cancer awaits further investigation.

The three Akt isoforms (Akt1, Akt2, Akt3) are encoded by different genes but they share a high degree of amino acid similarity [48]. We previously demonstrated a specific function of Akt3, but not Akt1 or Akt2, in TNBC growth [49]. By using a chronic resistance model of the breast cancer T47D line, we also identified Akt3 as an important mediator of Akt inhibitor resistance [16]. Here, we also observed de novo expression of Akt3 in BT474 resistant cells. Interestingly, this connects to a recently published work on Akt inhibitor resistance in castration-resistant prostate cancer (CRPC), where the authors also observed specific upregulation of Akt3, but not Akt1 or Akt2, in MK2206-resistance tumor cells [50]. However, in CRPC, the increased Akt3 levels are not reversible, whereas in our breast cancer model, upregulation of Akt3 involved an epigenetic mechanism [16]. Furthermore, overexpression or depletion of Akt3 in MK2206-resistant CRPC cells resulted in mild effects on Akt inhibitor sensitivity, and the authors concluded that Akt3 is likely to play a minor role in their resistance model. In contrast, they identified a point mutation (W80C) in Akt1 in MK2206-resistant cells and showed that this mutation participates in conferring resistance to MK2206 in CRPC cells. Therefore, individual Akt isoforms may have differential roles in conferring acquired resistance in different types of cancers.

In this study, we demonstrated an important role of pEGFR upregulation in Akt inhibitor resistance, and Gefitinib treatment may represent a potential therapeutic strategy for breast cancer patients with acquired resistance to Akt inhibitors. We further showed that the resistant cells acquired enhanced CSC properties, and our RNA sequencing uncovered several CSC-related genes that provide leads for further characterization. The ultimate goal of our study was to identify resistance mechanisms for designing combination strategies to overcome resistance, giving the moderate clinical effect of monotherapy of Akt inhibitors [4]. Indeed, using a similar chronic inhibitor treatment model, it was discovered that FOXO3a-BRD4-CDK6 signaling plays a key role in Akt inhibitor resistance in breast cancer [51]. This work and others have led to the current Phase Ib clinical trial on assessing the efficacy of a combination treatment of an Akt inhibitor and CDK4/6 inhibitor on breast cancer. It is clear that novel mechanistic insights for resistance mechanisms to the PI3K/Akt pathway targeted therapy will be essential for developing appropriate treatment regimens.

## Data Availability

All data in this study are available from the corresponding author on reasonable request.

## Supplementary Materials

The following supporting information can be downloaded at: https://www.mdpi.com/article/10.3390/cancers14205006/s1, Figure S1: Position of 49 different RTK antibodies on the membrane of phospho-RTK arrays; Figure S2: Reversibility of drug resistance to Akt inhibitor; Table S1: Genes differentially expressed in Akt inhibitor-resistant cells.
