# Supplementary material for: Upregulation of Receptor Tyrosine Kinase Activity and Stemness as Resistance Mechanisms to Akt Inhibitors in Breast Cancer
Source: Cancers (Basel). 2022 Oct 13;14(20):5006. doi: 10.3390/cancers14205006 (PMC9599323; doi:10.3390/cancers14205006)
Supplement: Supplementary file 1 [file cancers-14-05006-s001.zip › cancers-1892522-supplementary/cancers-1892522-supplementary figure.pdf]

B1<br>**EGFR**	B3<br>**ErbB2**	B5<br>**ErbB3**	B7<br>**ErbB4**	B9<br>**FGF R1**	B11<br>**FGF R2a**	B13<br>**FGF R3**	B15<br>**FGF R4**	B17<br>**Insulin R**	B19<br>**IGF1R**	B21<br>**Axl**	B23<br>**Dtk**
C1<br>**Mer**	C3<br>**HGF R**	C5<br>**MSP R**	C7<br>**PDGF Ra**	C9<br>**PDGF Rbet**	C11<br>**SCF R**	C13<br>**Flt-3**	C15<br>**M-CSF R**	C17<br>**c-Ret**	C19<br>**ROR1**	C21<br>**ROR2**	C23<br>**Tie-1**
D1<br>**Tie-2**	D3<br>**TrkA**	D5<br>**TrkB**	D7<br>**TrkC**	D9<br>**VEGF R1**	D11<br>**VEGF R2**	D13<br>**VEGF R3**	D15<br>**MuSK**	D17<br>**EphA1**	D19<br>**EphA2**	D21<br>**EphA3**	D23<br>**EphA4**
E1<br>**EphA6**	E3<br>**EphA7**	E5<br>**EphB1**	E7<br>**EphB2**	E9<br>**EphB4**	E11<br>**EphB6**	E13<br>**ALK**	E15<br>**DDR1**	E17<br>**DDR2**	E19<br>**EphA5**	E21<br>**EphA10**	
		F5<br>**EphB3**	F7<br>**RYK**								

Figure S1. Position of 49 different RTK antibodies on the membrane of phospho-RTK arrays. Each antibody spotted in duplicate.

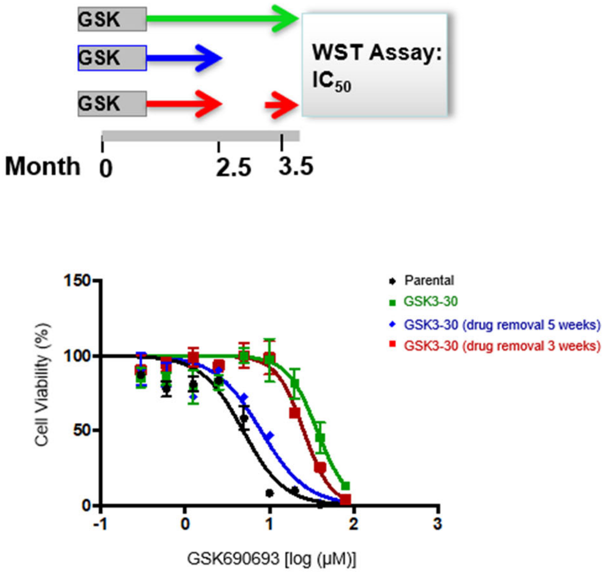

Figure S2. Reversibility of drug resistance to Akt inhibitor. To assess the ability of MDA-MB-231^{R} (GSK690693 3-30) cells to regain resistance after drug removal, a fraction of cells that have been cultured in the absence of drug for 3 weeks were re-challenged with GSK690693 for 2 weeks, followed by WST assays.
